# Intestinal Parasites and Fecal Cortisol Metabolites in Multi-Unowned-Cat Environments: The Impact of Housing Conditions

**DOI:** 10.3390/ani11051300

**Published:** 2021-04-30

**Authors:** Xavier Blasco, Xavier Manteca, Manel López-Béjar, Anaïs Carbajal, Joaquim Castellà, Anna Ortuño

**Affiliations:** 1 Animal Health and Anatomy Department, Veterinary Faculty, Universitat Autònoma de Barcelona, 08193 Bellaterra, Barcelona, Spain; xavi.blasco87@gmail.com (X.B.); manel.lopez.bejar@uab.cat (M.L.-B.); anais.carbajal@uab.cat (A.C.); joaquim.castella@uab.cat (J.C.); 2 Animal Science Department, Veterinary Faculty, Universitat Autònoma de Barcelona, 08193 Bellaterra, Barcelona, Spain; xavier.manteca@uab.cat

**Keywords:** cat, multi-cat environment, fecal cortisol, intestinal parasites, housing conditions

## Abstract

**Simple Summary:**

Multi-unowned-cat environments can be highly stressful for cats, and infectious and parasite diseases spread quickly and are difficult to prevent. This study aimed to determine the occurrence of intestinal parasites and fecal cortisol metabolites (FCM) in cat feces collected from different multi-cat environments and assess the effect of housing conditions on intestinal parasites and FCM levels in order to develop more efficient control strategies. Cat fecal samples from rescue shelters, catteries and feline colonies were analyzed with coprological methods to detect intestinal parasite patency and determine FCM. Helminth infection was mainly detected in free-roaming cats. In confined cats, protozoa infections were more likely detected in shelter cats than in cattery cats. Exposure to dogs was associated with parasite infection and cats highly exposed to dogs with visual contact and audible barking showed higher intestinal protozoa prevalence than cats that were not exposed to dogs. FCM levels were correlated with enclosure size and protozoa infection. Reducing stress by improving housing in terms of enclosure size and avoiding exposure to dogs may have an impact on the occurrence of intestinal parasites, especially protozoa.

**Abstract:**

Housing conditions were assessed in different unowned multi-cat management models in order to evaluate their impact on the occurrence of intestinal parasites and fecal cortisol metabolite (FCM) levels. Fresh stool fecal samples were collected from rescue shelters, catteries and feline colonies for coprological analyses in order to detect intestinal parasite patency and fecal cortisol metabolites. A questionnaire provided information about the facilities, management and housing conditions of cats, including information about dog exposure, enclosure size, environment enrichment and changes to group composition. Overall, intestinal parasite infection was detected in 58.2% of fecal samples collected. The occurrence of intestinal parasites detected in free-roaming cats was 82.2%, mainly due to helminth infection. The parasite infection rate was 57.3% in rescue shelters and 34.6% in catteries. In confined cats, protozoa infection was more likely detected in rescue shelters than in catteries (RR = 2.02 (1.30–3.14), *p* = 0.0012). Although the FCM values were very variable between cats, the enclosure size and parasite infection were correlated with the average FCM. A small enclosure size was correlated with high fecal cortisol metabolites (*p* = 0.016). Protozoa-positive samples showed higher FCM levels than negative samples (*p* = 0.0150). High dog exposure was statistically associated with protozoa infection (*p* = 0.0006). The results indicated that improving housing, especially in terms of floor space and avoiding dog exposure, reduces stress and can thus be applied to make control strategies in multi-unowned-cat environments more efficient, especially when cats are confined.

## 1. Introduction

The role of the domestic cat (*Felis catus*) as a pet has become more important in recent decades, but the number of unowned or owned–abandoned cats has also increased [1,2]. These cats are removed from the street and housed in different multi-cat environments, such as rescue shelters, catteries, sanctuaries, foster programs and feline colonies. Thus, some cats are sheltered under confined conditions until they can be rehomed or adopted, whereas feral cats are released into feline colonies constituted by a community of cats submitted to Trap–Neuter–Return (TNR) programs. Most of these multi-cat environments are managed by organizations that are usually working at full capacity, with very few economic resources.

Stray and shelter cats are highly exposed to intestinal parasites. Prevalence has been found to be 90% in Greece [3] and 77.4% in Switzerland in free-roaming cats [4], whereas in shelter cats, it ranged from 57% in Catalonia, Spain [5], 55.9% in Crete, Greece [6], 31.8% in Canada [7], 22% in Central Italy [8] to 21.8% in Switzerland [4]. *Toxocara cati*, *Giardia* sp. and *Cystoisospora* spp. constituted the most common parasites affecting free-roaming cats and shelter cats [3,4,6,8]. Some of these parasites, such as *Giardia* sp. and *Toxocara cati*, are responsible for zoonoses.

In addition to deworming, strategies for controlling intestinal parasites should be addressed at environmental conditions to minimize the spread of infection. Risk factors need to be identified to establish suitable control strategies for intestinal parasites. Different studies have documented the role of individual risk factors such as age, sex, immunological status and lifestyle (indoors/outdoors) in the transmission of intestinal parasites [4,9]; however, in cat populations, factors related to the environment, such as the management and housing conditions, should be taken into consideration since they could play an important role in the maintenance and transmission of parasites [5]. Thus, high animal density, poor hygienic conditions, environmental contamination, presence of asymptomatic carriers and poor housing facilities constituted important risk factors of intestinal parasites in animal shelters [10,11]. In addition, multi-cat environments can be highly stressful for cats, especially when unfamiliar cats are kept under confined conditions. In multi-cat households, environmental factors may play a more important role in controlling stress than the group size per se [12]. In unowned multi-cat settings, several stressor factors have been identified, including crowding, unsuitable housing, moving from one cage to another, introduction of new animals, lack of hiding areas, presence of dogs, group density and floor area [13,14,15,16,17]. Moreover, it should be pointed out that chronic activation of stress responses results in chronic production of glucocorticoid hormones, which interfere with immune cells and thus have an impact on immune response [18]. This could explain why cats with high stress scores are more likely to develop upper respiratory infection [17,19,20], which is highly prevalent in shelter cats [21]. The correlation between stress physiology and parasite infection has been widely reported in wildlife [22,23] and it has been suggested that the persistence of parasites in the host without inducing chronic stress responses supports the hypothesis that the hosts are tolerant to their parasites [24]. However, a study on dogs housed in an animal shelter revealed that, although it was suspected that shedding parasite ova would be impacted by the stressors associated with shelter housing, the plasma cortisol concentration decreased with time, whereas parasite shedding was unaffected by the duration of the shelter stay [25].

To the best of our knowledge, no studies have focused as yet on both intestinal parasites and fecal cortisol in cats. The main objectives of this study were to determine the occurrence of intestinal parasites (1) and detect the fecal cortisol metabolite levels in multi-cat environments (2), as well as to research the association, if any, between housing conditions, parasitism and fecal cortisol (3). Therefore, different management models of unowned multi-cat environments were studied: rescue shelters, where rescued pets (dogs and cats) are housed; catteries housing unowned cats exclusively; and feline colonies where free-roaming cats are captured to be included in the Trap–Neuter–Return (TNR) program. HOUSING conditions were assessed [14,26] and levels of fecal cortisol metabolites [27,28,29] were determined from feces that were also coprologically examined to determine the presence of helminth eggs, larvae and/or protozoan (oo)-cysts. It was hypothesized that free-roaming cats would have lower levels of cortisol than confined ones.

## 2. Materials and Methods

### 2.1. Study Population

A cross-sectional study was conducted in multi-cat environments located in Barcelona and Tarragona (Catalonia, Spain) and managed by private organizations. From March 2015 to October 2017, cat fecal samples were collected from rescue shelters, catteries and feline colonies. Multi-cat environment selection was based on no-kill policy settings that housed long-term adult cats. Fecal samples were collected from neutered, FIV/FeLV negative cats that were not dewormed for the last 3 months prior to sampling. With the exception of cats from feline colonies, the rest of the cats were waiting for adoption or rehoming. Sample collection was possible with previous authorization from shelters, catteries and municipalities in the case of feline colonies.

Fresh stool fecal samples were collected from the deposition tray or from the soil of cats housed inside. Samples were transported in a cooler to the Veterinary Parasitology Laboratory at the Veterinary Faculty and were stored at 4 °C. They were analyzed by coprological examination within 24 h after collection. The remainder was frozen at −80 °C for fecal cortisol metabolite assays.

A questionnaire, completed at the time of sampling, provided information about the facilities, management and housing conditions (Appendix A). Information about management included deworming and cleaning and disinfection protocols. Housing conditions were evaluated and included the following variables: enclosure size in terms of floor space per cat: >3 m^2^/cat; 1.67 m^2^/cat–3 m^2^/cat and < 1.67 m^2^/cat [13,14]; exposure to dogs:—no exposure (no dogs are housed in the same facility), low exposure (audible dog barking) and high exposure (visual contact) [15]; enriched environment (toys, scratching posts, hiding places, elevated shelves, etc.): provided, not provided; changes in group composition: low or high [26].

### 2.2. Fecal Score Test and Parasitology Analyses

Feces were macroscopically examined to detect adult stages or tapeworm proglottids. Applying the Fecal Scoring Test (FST) (Nestlé Purina Fecal Scoring System^®^), fecal consistency was classified as diarrhea or soft feces when the FST was ≥5 and normal or hard feces when the FST was ≤4. Coprological examination was carried out employing a previously described centrifugation flotation technique using zinc sulphate solution 33% (specific gravity 1.18 g/mL) flotation [30]. Parasite stages were identified using morphological keys [31].

### 2.3. Fecal Cortisol Metabolite (FCM) Detection

Fecal cortisol metabolites (FCM) and all the validation tests were determined by using cortisol enzyme immunoassays (EIA detection Kits, Neogen Corporation^®^, Ayr, UK). We followed the steroid extraction protocol (DetectXTM Steroid Extraction Protocol; Arbor Assays^®^, Michigan, MI, USA) with the modification suggested by other authors [32]. Samples were put in an oven at 37 °C for 48 h. Once dried, samples were manually pulverized with a mortar, and 300 mg of fecal powder was mixed with 2.5 mL distilled water and 3 mL methanol and vortexed for 30 min. After that, samples were centrifuged at 1008× *g* for 15 min and 1 mL of the supernatant was transferred into a 1.5 mL Eppendorf tube and stored at −80 °C until analysis.

Fecal cortisol metabolite quantification was based on standard solutions (1 µg/mL, 0.2 ng/mL, 2 ng/mL, 20 ng/mL). Briefly, the sample, the standard solutions and the diluted enzyme conjugate were added to the microplate and incubated at room temperature for one hour. The plate was then washed, removing all the unbound material. Later on, the substrate was added and incubated for 30 min. Quantitative test results were obtained by measuring and comparing the absorbance reading of the wells of the samples against the standards with a microplate reader at 650 nm.

#### Steroid Validation Test

For the validation of the assay, a fecal extract pool was created by mixing 20 samples of different concentrations. The precision within the test was assessed by calculating variation coefficients and intra- and inter-assay coefficients. The linearity of dilution was determined by using 1:1, 1:2, 1:5 and 1:10 dilutions of pools with EIA buffer. Accuracy was assessed through the spike-and-recovery test, calculated by adding volumes of 200, 100 and 50 μL of pure standard cortisol solution to 50, 100 and 200 μL of pool, respectively. According to the manufacturer, the cross-reactivity of the EIA antibody is as follows: prednisolone 47.4%, cortisone 15.7%, 11-deoxycortisol 15%, prednisone 7.83%, corticosterone 4.81%, 6β-hidroxicortisol 1.37%, 17-hidroxiprogesterona 1.36% and deoxycorticosterone 0.94%. Steroids with a cross-reactivity <0.06% were not included.

### 2.4. Statistical Analysis

Microsoft Excel 97–2003 for Windows (Microsoft Corporation, Redmon, WA, USA) and Epi-Info™ (v. 6.04d, CDC, Atlanta, GA, USA) were used to calculate the means and frequencies. The statistical analysis was performed using SAS^®^ (v. 9.4, SAS Institute Inc., Cary, NC, USA). The correlation between parasite infection rates, FST and housing conditions was calculated with the Chi-square test. Correlations between FCM and parasite infection rates and housing conditions were calculated with the non-parametric Kruskal–Wallis test. Differences were considered statistically significant when *p* < 0.05.

## 3. Results

### 3.1. Validation Test

The percentage recovery from the spike-and-recovery test was 78.31 ± 9.33%; the linearity of dilution showed a R^2^ = 99.07%, and the mean percentage of error was −6.33 ± 8.86%. The intra-assay coefficient of variation (cv) was 6% and inter-assay cv was 10.6%.

### 3.2. Sampling

A total of 368 cat fecal samples were collected from ten different multi-cat environments: three rescue shelters, three catteries and four feline colonies.

A total of 157 fecal samples were collected from three different rescue shelters where cats were living confined and were kept in large group housing with an escape-proof fence. Quarantine was applied to all cats at intake and they were dewormed and vaccinated. Deworming was based on a wide-spectrum anthelminthic in a single dose administered at intake and then every 4–6 months without any previous coprological examination. Feces were removed daily and disinfection was based on applying bleach (two shelters, *n* = 100) or a combination of bleach and ammonia (one shelter, *n* = 57). Facilities were built on concrete and feces were deposited on a cat litter tray. All of these rescue shelters also housed dogs; therefore, all cats from the rescue shelters were exposed to dogs and were exposed to either visual contact (one shelter, *n* = 31) or audible dog barking (two shelters, *n* = 126). Environmental enrichment (hiding places, scratching posts, toys, elevated shelves, etc.) was provided in all rescue shelters. In terms of the enclosure size, 38 fecal samples were collected from areas with floor space less than 1.67 m^2^ per cat, and 119 cat fecal samples came from areas where the floor space ranged from 1.67 to 3 m^2^ per cat (*n* = 119). New cats were introduced into the group as often as required and the turnover of cats was high in all the shelters in the study.

Cat fecal samples were collected from three different catteries (*n* = 104). All cats were living confined; they were kept in large groups and had access to an escape-proof fence. Quarantine was performed with all new cats at intake and they were dewormed and vaccinated. The deworming frequency was every 3–6 months. A wide-spectrum anthelminthic was given in a single dose without any previous coprological examination. Feces were deposited in a cat litter tray and they were removed daily. Disinfection was based on bleach and, in one cattery, it was combined with ammonia. Facilities were built on concrete. Environment enrichment was provided in all three catteries. No dog contact was documented. In terms of the enclosure size, cats were housed in rooms where the floor space ranged from 1.67 to 3 m^2^ per cat (*n* = 104). New cats were introduced whenever it was required, except for one cattery where the turnover of cats was low.

A total of 107 fecal samples were collected from four feline colonies constituted by free-roaming cats that were previously submitted to the TNR program. These cats were living under non-confined conditions. Cats were not dewormed. Fecal samples were collected from soil in the areas where feline colonies were established.

### 3.3. Fecal Score Test and Parasitology Analyses

Overall, intestinal parasite infection was detected in 58.2% (214/368) of fecal samples collected. The most frequent parasites detected were *Cystoisospora* spp. (20.6%, 76/368) and hookworms (18.2%, 67/368), which were not further identified. Prevalence of intestinal parasites was 82.2% (88/107) in feline colonies, 57.3% (90/157) in rescue shelters and 34.6% in catteries (36/104). The prevalence of intestinal parasites detected in each multi-cat environment sampled is listed in Table 1. Considering confined cats, protozoan infection was more likely detected in shelters than in catteries (*p* = 0.0012, RR = 2.02 (1.30–3.14, 95% confidence intervals). In feline colonies, helminth infection was the most prevalent (68.2%).

Infection by more than one parasite was detected in 103 fecal samples. Free-roaming cats hosted more than one parasite species to a greater extent (44.8%; 48/107) than shelter cats (28.6%, 45/157) and cattery cats (7.6%, 8/104). The most common coinfection in free-roaming cats was due to hookworms and lungworms. In cat shelters, coinfection was mainly due to *Giardia* spp. and *Cystoisospora* spp., whereas coinfection in cattery cats was due to protozoa and helminth infection.

A total of 100 fecal samples were classified as diarrhea or soft feces (FST ≥ 5). The remaining 268 fecal samples were classified as normal or hard feces (FST ≤ 4). Soft feces were mainly observed in shelters (33.8%, *n* = 53), whereas 25.2% (*n* = 27) of feces from free-roaming cats and 19.2% (*n* = 20) from catteries were soft. No statistical differences were detected when FST and parasite infection were compared.

### 3.4. Housing Conditions and Parasite Infection

Concerning housing conditions, statistical differences were observed when dog exposure and parasite infection were compared (*p* = 0.0055). This statistical association was also observed when protozoa infection and dog exposure were compared (*p* = 0.0006) as well as Giardia infection and dog exposure (*p* = 0.0010). Protozoa-positive samples were more likely detected in environments where exposure to dogs was high and there was visual contact and audible dog barking.

All shelter and cattery settings provided enriched resources to cat housing and this variable was not assessed in feline colonies since cats were living in conditions of freedom. No statistical differences were observed when comparing changes to group composition and parasite infection or enclosure size and parasite infection.

### 3.5. Fecal Cortisol Metabolite Values

Fecal cortisol metabolite (FCM) values ranged from 3.1 to 180.5 ng/g dry feces. The average of the FCM (±SD) values in samples collected in rescue shelters was 37.78 ng/g (±SD = 32.17); in catteries, it was 33.6 ng/g (±SD = 29.22), and in feline colonies, it was 28.13 ng/g (±SD = 16.15). No statistical differences were observed when comparing FCM and the multi-cat environments (*p* > 0.05).

### 3.6. FCM and Parasite Infection

Statistical differences were observed when average FCM and parasite infection were compared (K = 5.29; *p* = 0.0214). Protozoa-positive samples showed a higher average FCM (38.11 ng/g, ±SD = 30.28) than negative samples (31.73 ng/g; ±SD = 26.28) and this was statistically significant (K = 5.91; *p* = 0.0150). However, no statistical differences were observed when average FCM and helminth infection were compared (*p* > 0.05).

### 3.7. FCM and Housing Conditions

A correlation between enclosure size and average FCM was observed. Cats living in rooms where floor space was lower than 1.67 m^2^/cat showed higher average FCM (44.71 ng/g) than those living in rooms where floor space ranged from 1.67 to 3 m^2^/cat (34.687 ng/g) and those living in areas with >3 m^2^/cat (28.13 ng/g). Statistical differences were detected (K = 8.2; *p* = 0.0166).

The average FCM in cats with high exposure to dogs was 44.82 ng/g (SD = 38.99). It was 33.17 ng/g (SD = 26.61) in cats with low exposure to dogs, and in cats with no dog contact, it was 32.30 ng/g (SD = 26.07); however, no statistical differences were detected. Concerning changes to group composition, the average FCM was 28.52 ng/g (SD = 16.69) in highly stable groups and the average FCM was 36.31 ng/g (SD = 31.4) in environments where new cats were routinely introduced; no statistical differences were detected.

The average FCM was compared to variables related to parasite infection and FST (Table 2) and to welfare parameters (Table 3).

## 4. Discussion

Our results show that intestinal parasites are a major concern in unowned cats and may have an impact on animal and public health, because *Toxocara cati*, hookworms, *Dipylidium caninum* and *Giardia* sp. are considered potentially zoonotic.

Free-roaming cats were the most infected and they were mainly infected by helminths. Hookworms were the most frequent parasites detected. This is in agreement with other studies [4,33]. Most fecal samples from free-roaming cats were collected in soil, where cats buried their feces. In a previous study carried out by our research group, the highest prevalence of intestinal parasites was detected in samples deposited on soil [5]. Soil provides a suitable environment for parasites to develop into their infective stage, while protecting feces from desiccation, which is one of the mechanisms that is harmful to parasite survival [34]. However, lack of anthelminthic treatment and relevant hunting activity in free-roaming cats with ingestion of rodents or birds that may act as an intermediate or paratenic host in the biological cycle of most of these helminths may explain this result.

Although shelter and cattery cats were regularly dewormed, the results of the parasite infection rate detected in both groups of confined cats were not as good as expected. Deworming was based on wide-spectrum anthelminthic treatment in a single dose, which could be a good strategy to control helminth infection but not protozoa infection [35,36]. According to ESCAAP [37], it is recommended that deworming takes place once a month or fecal samples are examined every four weeks and treated according to findings for cats kept in shelters or catteries or free-roaming cats. However, deworming was performed without any previous coprological examination, leading to blind treatments and incorrect deworming schedules [36]. Since no statistical association was observed when the fecal score (normal, soft or diarrhea) was compared with parasite infection, it could be assumed that most cats that hosted intestinal parasites were healthy and this strongly suggests the role of newly entered asymptomatic cats as a source of infection in the community, which is in accordance with previously reported studies [38,39].

It was observed that the average FCM was higher in those samples that were positive for protozoa (oo)-cysts. As has been suggested by other authors [23], there is a complex interaction between host stress physiology, immune function and parasite infection. The most important immune response against micro-parasites (i.e., protozoan parasites) is the Th1 response, whereas helminth infection activates the Th2 pathway [40], and cortisol and other neuroendocrine HPA axis mediators can shift the immune response from Th1 to Th2 [18]. Higher FCM may have favored the Th2 response, which may be responsible for the decreased Th1 response, leading to high protozoa prevalence in those communities with a high FCM level. This could explain why protozoa-positive fecal samples showed the highest fecal cortisol levels, whereas no statistical association was observed when average FCM was compared with helminth infection.

Confinement may favor the maintenance and transmission of protozoa infection since environmental contamination and overcrowding may constitute important risk factors [8,39]. Apart from free-roaming cats, the confinement and management model were similar in rescue shelters and catteries; however, protozoa infection was more likely detected in shelter cats. The large difference between rescue shelters and catteries could be related to dog exposure (audible barking noise and even visual contact). Interestingly, a positive correlation was observed between high dog exposure and protozoan infection rate. According to previous studies [15], the largest factor affecting the cat’s stress levels appeared to be the extent of exposure to dogs. Although our results did not show any association between dog exposure and FCM levels, this could be due to the low number of cats subjected to high dog exposure.

In our study, all confined cats were housed in groups and results showed a correlation between floor space per cat and average FCM. Cats need more floor space when housed in groups than when housed individually in order to prevent conflict and to compensate the additional stress caused by unfamiliar group members during the first two weeks of stay [26,41]. In our study, none of the cats surveyed had been recently introduced into the setting because all of them were neutered cats and regional legislation states that unowned cats could not be neutered before three weeks after intake.

Most of the cortisol metabolites in cats are excreted via the feces [42]. Determination of the fecal cortisol metabolite is a non-invasive approach for monitoring physiological responses to stress. Moreover, fecal samples can be collected easily without handling the animal. EIA was a useful technique for determining the cortisol level in carnivorous species [28] and the validation test using this methodology was carried out successfully. The FCM concentration is the result of the HPA axis activity that occurred previously (6–24 h) [43]. However, in line with other studies [12,40], a large inter-animal variation in fecal-cortisol metabolite concentrations was observed and there was a lack of a normal reference range—for instance, in non-stressed cats. Cats included in this study were subjected to a single FCM measurement, which is not representative of the chronic stress response; however, since fecal samples could be analyzed by both coprological techniques as well as EIA for cortisol metabolite analyses, our results could represent an approach for the eventual association between cortisol level and parasite infection in different multi-cat management models, as an alternative to an individual cat approach.

To the best of our knowledge, this is the first study dealing with intestinal parasite patent infection and FCM in unowned cats. However, some limitations may have affected the outcomes of this study. In agreement with other authors [25], there are several challenges involved in researching a multi-unowned-cat environment as it is not possible to control unknown backgrounds and there are different confounding variables directly associated with the housing model. Hence, individual cats could not be monitored over a given period of time because of the turnover of animals. Furthermore, fecal samples collected from feline colonies may include stray cats that could be uncontrolled. Furthermore, the individual temperament and past experiences of surveyed cats should be taken into consideration [44]. Furthermore, it was suggested that cats spending longer periods of time in the shelter may develop signs of depression that may cause low fecal cortisol levels; conversely, in shelters with environments more conductive to the cats’ needs, the physiologic measure of stress decreased with time [15]. It has been found that cats can adapt to new environments in 2–5 weeks, but this shows great individual variation among cats [13]. Another limitation concerns the flotation concentration technique. This is a good technique for routine coprological diagnosis; however, some protozoal infections, such as *Tritrichomonas foetus*, cannot be detected with this technique and the real intestinal parasite prevalence could be higher than that detected. Finally, parasite excretion varies dramatically within and between individuals, and parasite intensity in any given fecal sample may not directly correlate with the parasite burden in the host.

Most shelter or cattery cats are usually waiting for adoption, rescue or reclamation by owners and stray cats can act as a source of soil contamination; it is necessary to implement suitable control strategies to improve the health status in multi-cat environments and reduce the risk of zoonosis. Implementing environmental strategies to reduce stress, especially improving housing and avoiding exposure to dogs, may have an impact on protozoa infection, especially in confined cats. Further studies are necessary to clarify the role played by stress in the prevalence of the most common intestinal protozoa affecting cats.

## 5. Conclusions

Intestinal parasites were highly prevalent in unowned cats. When parasite infection was compared in different multi-unowned-cat management models, it was observed that free-roaming cats were mainly infected by helminths, whereas in confined cats, protozoan infection was more likely detected in shelter cats than in cattery cats. Although the FCM values were very variable among cats, enclosure size was correlated with the average FCM. Protozoa-positive samples showed higher FCM on average than negative samples and were more likely detected in environments with high dog exposure. The results suggest that improving housing, especially in terms of floor space and avoiding dog exposure, can be used to make control strategies more efficient, especially in confined cats.

## Figures and Tables

**Table 1:** Prevalence of intestinal parasites detected in samples collected from rescue shelters, catteries and feline colonies using a flotation concentration coprological technique.

Multi-Cat Environments	Rescue Shelters	Catteries	Feline Colonies	Total
Cat fecal samples (*n*)	*n* = 157	*n* = 104	*n* = 107	*n* = 368
Parasites	*n* (%)	*n* (%)	*n* (%)	*n* (%)
Protozoa	61 (38.8%)	20 (19.2%)	39 (36.4%)	120 (32.6%)
*Giardia* spp.	35 (22.3%)	13 (12.5%)	14 (13.1%)	62 (16.8%)
*Cystoisospora* spp.	39 (24.8%)	8 (7.7%)	29 (27.1%)	76 (20.6%)
Helminths	48 (30.5%)	21 (20.2%)	73 (68.2%)	142 (38.6%)
Tapeworms	16 (10.2%)	8 (7.7%)	24 (22.4%)	48 (13%)
*Toxocara cati*	21 (13.3%)	4 (3.8%)	20 (18.7%)	45 (12.2%)
*Toxascaris leonina*	0	1 (0.9%)	0	1 (0.2%)
Hookworms	11 (7%)	4 (3.8%)	52 (48.6%)	67 (18.2%)
Metastrongylidae	11 (7%)	8 (7.7%)	21 (19.6%)	40 (10.8%)
*Capillaria* sp./*Eucoleus* sp.	0	0	5 (4.6%)	5 (1.3%)
Total	90 (57.3%)	36 (34.6%)	88 (82.2%)	214 (58.2%)

**Table 2:** FCM average and variables related to parasite infection and FST (* significance level *p* < 0.05).

	Fecal Cortisol Metabolite (ng/g)				
**Variable/Category**		**N**	**Average**	**SD**	***p*-Value**
Parasite infection	+	214	34.29	25.17	0.021 *
	−	154	33.14	31.09	
Protozoa infection	+	120	38.11	30.28	0.0150 *
	−	248	31.73	26.28	
Giardia infection	+	62	40	32.09	0.056
	−	306	32.56	26.69	
Helminth Infection	+	142	31.71	21.99	0.905
	−	226	35.13	30.82	
Fecal score test (FST)	≥5	100	33.94	22.72	
	≤4	268	33.77	29.47	

**Table 3:** FCM average and variables related to welfare parameters (* significance level *p* < 0.05).

	Fecal Cortisol Metabolite (ng/g)					
**Welfare Parameters in Housing Conditions**		Multi-Cat Environment (*n*) **	**N**	**Average**	**SD**	***p*-Value**
Floor space	<1.67 m^2^/cat	Rescue shelters (*n* = 38)	38	44.71	35.99	0.016 *
	1.67–3 m^2^/cat	Rescue shelters (*n* = 119) Catteries (*n* = 104)	223	34.68	29.96	
	>3 m^2^/cat	Free-roaming cats (*n* = 107)	107	28.13	16.15	
Dog exposure	Visual contact	Rescue shelters (*n* = 31)	31	44.82	38.99	0.093
	Audible barking noise	Rescue shelters (*n* = 126) Feline colonies (*n* = 68)	194	33.17	26.61	
	No dogs	Catteries (*n* = 104) Feline colonies (*n* = 39)	143	32.30	26.07	
Changes to group composition	High turnover	Rescue shelters (*n* = 157) Catteries (*n* = 93)	250	36.31	31.40	0.338
	Low turnover	Feline colonies (*n* = 107) Cattery (*n* = 11)	118	28.52	16.69	

** (*n*) = number of cat fecal samples.

## Appendix A. Questionnaire

**Table:**

Animal ID				
	Date of sample collection		Location	
	Age			
Housing model				
	Rescue shelter	Cattery	Feline colony	
	N animals/setting			
Samples				
	FST			
	Coprological results		Parasites detected	
Management				
	Deworming	Y	N	
	Drug			
		Oral	Spot-on	Other (specify)
	Frequency			
	Date of last deworming			
	Any coprological control?	Regular	Occasional	Never
		If yes, results:		Date
				
	Environment disinfection protocol	Product		
		Frequency		
Housing conditions				
	Floor space	>3 m^2^/cat	1.67–3 m^2^/cat	<1.67 m^2^/cat
	Exposure to dogs	High	Low	No contact
	Environmental enrichment	Provided	No provided	
	Changes in group composition	Frequent	Low	
Facilities				
	Box	Number of animals/box		
	Floor material	Concrete	Soil	Other (specifiy)
	Tray material	Litter	Soil	Other (specifiy)
